# Editorial: Neuropeptides and Behavior: From Motivation to Psychopathology

**DOI:** 10.3389/fendo.2017.00210

**Published:** 2017-08-25

**Authors:** Deborah Suchecki, Carol F. Elias

**Affiliations:** ^1^Departamento de Psicobiologia, Escola Paulista de Medicina, Universidade Federal de São Paulo, São Paulo, Brazil; ^2^Department of Molecular and Integrative Physiology, University of Michigan, Ann Arbor, MI, United States

**Keywords:** neuropeptides, behavior, psychiatric disorders, sex differences, neuronal plasticity

**Editorial on the Research Topic**

**Neuropeptides and Behavior: From Motivation to Psychopathology**

The discovery of the involvement of neuropeptides with behaviors other than regulatory motivated ones took place in the mid-1960s with David de Wied’s first report on the influence of stress hormones, such as ACTH and arginine vasopressin (AVP) infused intra-cerebroventricularly, on memory (1). This major scientific breakthrough opened a new frontier of studies in Endocrinology and its related fields, Neuroendocrinology and Psychoneuroendocrinology.

Neuropeptides were thought to be involved in homeostatic regulation and secreted only from hypothalamic neurons; they are now recognized neurotransmitters, produced in, and secreted from distinct brain areas, associated with a myriad of, not only motivated but also psychopathological behaviors (2). Motivated behaviors are determinant for individual and species survival, but their expression in a large spectrum and deviations from average may give rise to a number of psychiatric conditions.

This research topic brings together 3 original studies and 10 review papers on the regulatory role of neuropeptides on sleep, feeding-, maternal-, and social behaviors. In addition, the implication of neuropeptides on both ends of this spectrum—lack of or excessive motivation—will also be discussed, as more and more, changes in neuropeptide production such as neuropeptide Y (NPY), oxytocin (OT), corticotropin-releasing hormone/factor (CRH/CRF), orexin, melanin-concentrating hormone (MCH), prolactin, and opioids have been associated with depression, anxiety, drug addiction, eating, and social disorders.

Pomrenze and colleagues used a transgenic rat that expresses CRH immunoreactive neurons, and they were able to show that this rat is a useful tool to study CRH projections, mainly from the central nucleus of the amygdala and the bed nucleus of the *stria terminalis*. CRH is the primary mediator of the hypothalamic–pituitary–adrenal axis stress response, subjected to plasticity and adaptations in cases of chronic stress exposure. Herman and Tasker presented a review of the mechanisms involved in the adaptations of the stress response at the level of the paraventricular nucleus of the hypothalamus, including the enhancement of CRH and AVP production, reduced glucocorticoid negative feedback activity, and increased excitatory neurotransmission at the CRH neurons. These converging alterations lead to increased CRH activity, characteristic of a number of stress-related psychiatric disorders.

In recent years, dysregulation of extra-hypothalamic CRF systems has gained attention in the context of alcohol use disorders. Quadros and colleagues presented a comprehensive review on the role of CRF and urocortin systems (and their related elements), in alcohol-induced escalated aggression, and in the transition from alcohol use to dependence, due to the direct modulation of CRF on the brain reward system.

In mice, chronic administration of low doses of ethanol produce a stimulatory action, inducing a phenomenon known as behavioral sensitization, which reflects neurobiological modifications in the reward system. Kawakami and co-workers showed that expression of behavioral sensitization can be blocked by naltrexone, an antagonist of the μ and, to a lesser extent, of the κ opioid receptors, in a sex-dependent manner, with greater efficiency in males than in females.

Keil and colleagues explored the involvement of the cyclic AMP/protein kinase A (cAMP/PKA) signaling pathway in anxiety and depressive disorders, by means of amygdala hyperactivation induced by stressful conditions. PKA is involved in the regulation of a myriad of intracellular signals triggered by distinct neurotransmitter systems related to alertness, mood, and acute and social anxiety. This mechanism of plasticity could be important for the proposition of new therapeutic strategies for conditions that afflict the human population.

Stress recruits numerous neuromodulators and neuropeptides that provide optimal coping conditions and adaptive responses. One such response is increased sleep time, especially of REM sleep, after a stressful event. Machado and Suchecki reviewed the neuropeptidergic and hormonal mechanisms involved in sleep induced by stressful situations. In Wellman and coworker’s original article, escapable shock, a model of controllable stress, increased REM sleep, whereas inescapable shock reduces this sleep stage. CRF blocked this positive response to controllable stress, whereas astressin, a CRF receptor 1 antagonist, restored sleep to control levels.

Melanin-concentrating hormone was the focus of Torterolo and colleagues’ review with special emphasis on recent electrophysiological recordings and optogenetic stimulation. The authors argued that the greatest firing rate of MCHergic neurons occurred during REM sleep and remote stimulation of those neurons induced sleep. The authors also focused on MCH projections to the dorsal raphe nucleus and the potential role of MCH in the pathophysiology of depression.

Neuropeptide Y and its receptors have a key role in central control of energy homeostasis, sleep, circadian rhythm, memory, and neuronal plasticity. Thorsell and Mathé presented an overview of NPY action on anxiety and stress response, and potentially post-traumatic stress disorder and depression. The authors discussed genetic and epigenetic findings of importance for NPY function and regulation and proposed that the modulation of NPY-ergic activity within the brain constitute potential targets for intervention in affective and alcohol use disorders.

Circulating hormones are also key players in motivation and behavioral adaptations. Among the most versatile of them is prolactin (PRL). Torner highlighted the role of PRL in neurogenesis and stress responses in the male brain. The author showed strong argument supporting the hypothesis that alterations in the PRL system due to stress or exposure to substances or other conditions that reduce neurogenesis may contribute to maladaptive responses and pathological behavioral outcomes. The author discussed the effects of PRL on neurogenesis and neuroprotection and their potential contribution to the onset of psychopathological states such as depression.

Oxytocin is a neuropeptide synthesized primarily by neurons of the paraventricular and supraoptic nuclei of the hypothalamus. These neurons are part of the magnocellular neurosecretory system, releasing OT into the posterior pituitary to promote labor and lactation. However, OT neurons also project to other brain sites and regulate homeostatic processes, social recognition, and fear conditioning. OT also decreases neuroendocrine stress signaling pathway, anxiety, and depression-like behaviors. Acevedo-Rodriguez and colleagues presented data showing that steroid hormones differentially modulated stress responses and altered OT receptor expression. The authors emphasized the role of estrogen receptor β activation and suggested a role for OT in this estrogen receptor β-mediated anxiolytic effect.

The function of neuropeptides and hormones is modulated by neuronal plasticity, synaptic strength, and morphological changes. Cabral and colleagues focused on the importance of mechanisms associated with the permeability of the blood brain barrier (BBB) and the interplay among delicate anatomical interfaces that have the potential to alter physiology and behavior. Authors used ghrelin, a hormone produced by the stomach with important role in energy homeostasis and motivated behaviors. Ghrelin receptors are widespread in the brain, but the accessibility of the hormone is strikingly low. This neuroendocrine issue is discussed in light of the dynamic control of the BBB.

Another aspect discussed in this issue is the crucial role of synaptic strength and interaction in physiology and behavior. Woelfle and colleagues discussed the effect of TCAP (short amino acid sequence found in the distal extracellular tip of teneurin) and the LPHN ligand–receptor system in anxiety, stress, and mood disorders. The teneurin TCAP protein binds and activates the LPHNs, a family of adhesion-associated GPCRs, to regulate numerous neurological and physiological activities. The long-lasting effect of exogenous TCAP and the current molecular model of ligand–receptor interaction suggest a role in neuronal plasticity and behavioral modulation.

Understanding the interplay between neuropeptides and hormones in the control of motivation and behavioral responses is highly relevant for human’s physical and mental health. Clearly, research in this field is advancing at a rapid pace. The articles in this eBook highlight novel findings and unanswered questions for future investigation.

## Author Contributions

DS and CE participated as editor of 5 papers, each, of the 13 published in this Research Topic. They jointly wrote the present editorial.

## Conflict of Interest Statement

The authors declare that the research was conducted in the absence of any commercial or financial relationships that could be construed as a potential conflict of interest.

## References

[1] De Wied D The influence of the posterior and intermediate lobe of the pituitary and pituitary peptides on the maintenance of a conditioned avoidance response in rats. Int J Neuropharmacol (1965) 4:157–67. 10.1016/0028-3908(65)90005-5 14342019

[2] Belzung C Yalcin I Griebel G Surget A Leman S. Neuropeptides in psychiatric diseases: an overview with a particular focus on depression and anxiety disorders. CNS Neurol Disord Drug Targets (2006) 5:135–45. 10.2174/187152706776359682 16611088

